# An Integrative Tinnitus Model Based on Sensory Precision

**DOI:** 10.1016/j.tins.2016.10.004

**Published:** 2016-12

**Authors:** William Sedley, Karl J. Friston, Phillip E. Gander, Sukhbinder Kumar, Timothy D. Griffiths

**Affiliations:** 1 Institute of Neuroscience, Newcastle University Medical School, Newcastle upon Tyne, UK; 2 Wellcome Trust Centre for Neuroimaging, University College London, London, UK; 3 Human Brain Research Laboratory, University of Iowa, Iowa City, IA, USA

**Keywords:** tinnitus, precision, predictive coding, auditory cortex

## Abstract

Tinnitus is a common disorder that often complicates hearing loss. Its mechanisms are incompletely understood. Current theories proposing pathophysiology from the ear to the cortex cannot individually – or collectively – explain the range of experimental evidence available. We propose a new framework, based on predictive coding, in which spontaneous activity in the subcortical auditory pathway constitutes a ‘tinnitus precursor’ which is normally ignored as imprecise evidence against the prevailing percept of ‘silence’. Extant models feature as contributory mechanisms acting to increase either the intensity of the precursor or its precision. If precision (i.e., postsynaptic gain) rises sufficiently then tinnitus is perceived. Perpetuation arises through focused attention, which further increases the precision of the precursor, and resetting of the default prediction to expect tinnitus.

## Trends

Existing tinnitus models, including mutually exclusive mechanisms, invoke causes from the ear to high-level cortical brain networks.

The generic framework of predictive coding explains perception as the integration of sensory information and prior predictions, each weighted by its precision.

In our model, previously proposed neural correlates of ‘tinnitus’ largely relate to hearing damage, rather than to tinnitus *per se*, and reflect an increase in the precision of spontaneous activity in the auditory pathway, which acts as a tinnitus precursor.

Perception of tinnitus emerges if the precision of the precursor rises sufficiently to override the default (null hypothesis) percept of ‘silence’.

Tinnitus becomes chronic when perceptual inference mechanisms learn to expect tinnitus, engaging connections between auditory and parahippocampal cortex.

## Why Understanding Tinnitus Matters

Fourteen percent of adults experience chronic tinnitus [1], while over 50% of normal-hearing adults experience subtle ongoing tinnitus within a silent environment 2, 3. Hearing loss is the biggest risk factor, followed by increasing age [1]. No widely applicable treatment reliably suppresses or eliminates tinnitus; in part, this is due to incomplete understanding of underlying pathophysiology. Improved understanding might also help clinicians to explain the condition to patients, and offer a unique window into sensory processing – without the confounding effects of an external stimulus. Furthermore, tinnitus may share commonalities with other aversive sensory conditions such as chronic pain 4, 5.

## The Symptomatology and Pathophysiology of Tinnitus

Tinnitus is the experience of persistent sound, in one or both ears or inside the head, in the absence of an external source [6]. In ‘objective’ tinnitus there is a measurable internal sound source such as turbulent blood flow, while the majority of tinnitus cases are ‘subjective’, where no such source exists. Tinnitus is perceived as fairly quiet, often masked by sufficient levels of environmental sounds, but a minority of cases are reported as extremely loud, and some are exacerbated by environmental sound [7]. Sounds are usually simple, with common forms resembling pure tones (‘ringing’), Gaussian noise (‘hissing’), or buzzing. More complex sounds are reported, and a minority of cases comprise music, for which we have recently proposed a related but distinct brain model to the tinnitus model described here [8]. Most people experience transient tinnitus at times, either spontaneously or following loud or prolonged noise exposure. Once tinnitus has been present for weeks to months, unless a reversible cause of hearing impairment is present, it typically becomes permanent. While it does not usually resolve spontaneously, the natural history tends to be of **habituation** (see Glossary) over time. However, a minority of patients report increasingly severe symptoms [7].

Existing evidence on the physiological basis of tinnitus is extensive and wide-ranging, but several paradoxes remain unsolved:

(i) Although hearing loss is its major risk factor, tinnitus only accompanies some cases, and often occurs at a later time, such as during physiological or psychological stress [9]. Furthermore, tinnitus can occur irrespective of the severity of hearing loss 10, 11, and more than half of normal-hearing adults experience slight tinnitus if placed in a sound-proof room 2, 3.
(ii) While most clinically significant tinnitus follows damage to the auditory **periphery**, which reduces afferent input and leads to increased **central** gain via homeostatic mechanisms [10], tinnitus cannot be explained solely by such a ‘central’ model, nor by a purely peripheral one (Box 1). Initially after acoustic trauma, spontaneous central auditory firing is correlated to peripheral (cochlear) firing [12], and during this phase pharmacological suppression of cochlear activity eliminates behavioural evidence of tinnitus [13]. Nevertheless, cochlear suppression in humans via chronic ear plugging has been shown to **cause** tinnitus [14]. In chronic tinnitus patients, pharmacological cochlear suppression is only sometimes effective [15], and sectioning the auditory nerve can alleviate or exacerbate tinnitus [16].
(iii) While many human and animal studies comparing hearing-impaired tinnitus subjects to normal hearing controls have found altered spontaneous neural activity patterns in the auditory pathway 17, 18, 19, 20, 21, the minority of such studies that controlled for hearing loss have not replicated these findings [18]. Therefore, such neural changes are probably related to hearing loss rather than to tinnitus itself, and tinnitus presently lacks a distinguishing neural correlate.
(iv) Arguably the closest neurophysiological correlates of tinnitus in humans are auditory cortex delta/theta and gamma band **oscillations** 17, 18, 21, 22, 23, 24, 25, 26, 27, 28, 29. However, neither of these consistently reflects perceived tinnitus intensity during short-term modulations that follow acoustic stimulation 18, 26, 27, 29: in **residual inhibition (RI)** 26, 30, where tinnitus is reduced, both delta/theta and gamma are suppressed (i.e., positive correlations); in **residual excitation (RE)** [26], where tinnitus is increased, delta/theta is unchanged and gamma is reduced (i.e., negative correlation). Therefore, neither oscillation can simply be a correlate of ‘tinnitus’, nor can gamma oscillations be a signature of sensory change [31] because, in that case, they would increase during either type of tinnitus modulation rather than reduce. Furthermore, these differential correlations cannot be due to inter-subject differences because the dichotomy is present within the same individuals.

Existing models propose a range of origins and mechanisms of tinnitus generation, involving pathophysiology in the peripheral [12] or central [32] auditory pathways, or in higher perceptual networks [33]. However, none of these, alone or in combination, can explain all the paradoxes described above. Furthermore, most of these models are mutually incompatible (e.g., if a case of tinnitus is due to increased ascending activity in the auditory brainstem [10], then it cannot simultaneously be due to reduced ascending brainstem activity leading to thalamic hyperpolarisation 31, 34). Box 1 discusses existing models in more detail.

This article addresses the ‘hard problem’ of tinnitus (i.e., how the sound itself is generated and perceived) and leaves aside other important issues such as emotional, cognitive and autonomic reactions. Its scope is to introduce a framework that can account for empirical evidence and known tinnitus phenomenology, settle unsolved paradoxes, and incorporate existing theories as complementary routes into a common mechanism. Our treatment is divided into three sections that explain the model in conceptual, neurobiological, and computational terms. As a prelude, we introduce the concept of **predictive coding**, upon which our framework is built.

## Basics of Predictive Coding

Predictive coding 35, 36, 37 assumes that sensory systems are organised hierarchically, and each hierarchical level contains state units (neurons) which encode representations of environmental states. These units generate **predictions** of states in the level below, with the nature, scope, and complexity of the implicit representations being determined by the hierarchical level. Sensory states occupy the lower level of the hierarchy. Each state unit has an associated error unit that encodes the difference between the expected state and its prediction from the level above. Error units send **prediction errors** to the level above, enabling state units to provide better predictions of the level below. This induces reciprocal message-passing with ascending prediction errors and descending predictions. At the lowest level (sensory epithelia), sensory input generates a sensory prediction error. At higher levels, **prior** prediction errors report the mismatch between expected state of the world (at that hierarchical level of abstraction) and top-down predictions. Prediction errors therefore drive expectations and, when there is complete congruence of hierarchical predictions and sensory input, there are no ascending prediction errors. This means that observable neuronal activity is a signature of disequilibrium, rather than of perception *per se*.

From a statistical perspective, state units encode **posterior** beliefs, which are the product of sensory input, in the form of a **likelihood**, and a prior from the level above. In predictive coding, each belief entails a Gaussian probability distribution over a perceptual dimension such as the intensity of tinnitus. The inverse variance of each distribution is its **precision**, which corresponds to the confidence placed in that belief 36, 38. The precision-weighted mean of the prior and likelihood is the posterior expectation that encodes the most likely value within the perceptual dimension (the inset in Figure 3, below, for givesgives an illustration of this process of inference). The posterior becomes the likelihood for comparison with the prior at the next hierarchical level above, and influences the prior with respect to the next level below. The likelihood and prior compete to update the posterior expectation, in proportion to their precision. In terms of neural dynamics this means that posterior expectations are driven by precision-weighted prediction errors (PWPE). Precision is thus crucial in representing uncertainty and making optimal use of available information from multiple sources.

Based on known neuronal microcircuits [39], simulations, and empirical observations 40, 41, 42, 43, 44, 45, 46, predictions and prediction errors have been linked to oscillations in specific frequency bands. State units are thought to be located in infragranular (deep) layers, and generate predictions using beta and other low-frequency oscillations. Error units occupy supragranular (superficial) layers, and communicate prediction errors using high-frequency gamma oscillations. While proponents of predictive coding may claim that all instances of each of these oscillation types can be understood in this framework, there have been many other proposed roles – in the case of gamma oscillations ranging from conscious perception [47], through inhibition [48], to generic signatures of activation [49]. Time may tell whether a single framework accounts for this diverse array of spectral correlates.

The fundamental encoding of precision is conceptually straightforward; superficial pyramidal neurons receive excitatory input, representing the bottom-up likelihood, and inhibitory input representing the top-down prediction. Where these two inputs are congruent their postsynaptic potentials cancel, and where they are not a prediction error is generated [39]. Precision is the postsynaptic gain that scales that prediction error [36], modulating its influence on higher levels. Postsynaptic gain at any cortical level is influenced by neuromodulators including acetylcholine 38, 50, which is released through activation of the basal forebrain system and mediates the effects of attention, memory, and learning. This provides a dynamic, context-sensitive, mechanism for adaptively modulating precision (e.g., direction towards internal prior representations during thought or imagery, and towards external stimuli during focused attention). Where a neuron receives multiple inputs, the synchrony of the excitatory postsynaptic potentials that are induced is an important determinant of precision because synchrony influences temporal summation and thereby the chance of crossing the threshold necessary for depolarisation. This phenomenon is termed synchronous gain, and is particularly sensitive to synchrony within the gamma band 51, 52. A role for lower-frequency oscillations is much less well established; recent empirical evidence has linked low-frequency oscillation magnitude to the precision of internal representations [41] including, in the case of alpha oscillations, the precision of the prediction that a sensory change will not occur [53]. This latter claim has challenged the widely-held view that alpha oscillations simply modulate cortical excitability. However, a mechanism linking low-frequency oscillations to precision is less apparent. Synchronous gain would be much weaker under such long timescales, but low-frequency oscillations are known to organise high-frequency oscillations [54], create temporal windows for stimulus processing, and allow multiplexing (simultaneous representation of multiple objects within the same neuronal ensembles by segregation in time) [55], and also show long-distance coherence, particularly in tinnitus [27] – and as such may exert indirect effects on precision or exert a complementary effect on the transmission of sensory information.

While precision is not directly encoded in stimulus properties, these are nonetheless highly relevant. For instance, a noisy stimulus leading to irregular neuronal firing would be relatively unpredictable at hierarchical levels relevant to the processing of short-timescale stimulus-intensity fluctuations. The low predictability would lead to irreducible prediction errors, that inherently entail a reduction in **sensory precision**, and a complementary increase in the precision afforded to higher expectations. The timescales of firing-rate integration likely increase with hierarchical level.

The encoding of stimulus intensity or its perceptual correlate (loudness, in the auditory modality) is not fully understood. Evidence suggests that, below the level of auditory cortex, neuronal activity relates to stimulus intensity, while at a cortical level it represents perceived loudness [56]. However, it is not known to what extent the subsequent representation of loudness is maintained parametrically through neuronal firing rate, or to what extent it is abstracted as in other auditory dimensions such as pitch [57]. However, it is clear that intensity is not simply precision because mismatch responses, which represent violation of predictions, are reliably elicited to positive and negative changes in stimulus intensity [58]. Irrespective of how it is encoded, the representation of loudness is probably modulated by precision as with other perceptual attributes.

## The Conceptual Model

Because all sensory systems are continuously active, there is always a spontaneous prediction error, which in the auditory system can be considered as a tinnitus precursor. This precursor has inherently low precision owing to its noise-like stimulus properties, an adaptive reduction in sensory precision resulting from deafferentation, and its lack of behavioural relevance (i.e., its inability to predict other stimuli or events). By default, prior predictions of auditory input are either of silence or the consequences of auditory objects. As precision of the tinnitus precursor is generally lower than that of the prior prediction against which it is compared, it has a negligible impact on perception, except in unusually quiet environments during focused attention 2, 3. The key insight here is that the percept of ‘silence’ is the brain's explanation for a particular pattern of imprecise prediction errors normally encountered in the absence of structured auditory input. However, this null hypothesis of ‘silence’ may be rejected if the tinnitus precursor reaches a sufficient intensity and/or is afforded too much precision. Respectively, intensity and precision of the precursor are akin to the mean and variance in a *t* test, and together determine the statistical significance of its deviation from the null hypothesis.

Factors predisposing to tinnitus act, alone or synergistically, in one of two broad and complementary ways: either they increase the intensity of the tinnitus precursor by increasing subcortical firing rates (e.g., via increased central gain [10]), or they increase the sensory precision of the tinnitus precursor. We posit that neural correlates so far attributed to ‘tinnitus’ (such as gamma oscillations in auditory cortex 17, 22, 23) are in reality largely correlates of increased precision of the tinnitus precursor or latent prediction error. This explains why these correlates have not been found to differ between tinnitus subjects and equivalently predisposed controls (e.g., [18]). If the intensity or precision of the precursor rises sufficiently (e.g., due to acute deafferentation or intercurrent stress, respectively), or its attenuation is insufficient, then it influences perception, and ‘tinnitus’ supervenes over the percept of ‘silence’.

**Figure 1:**
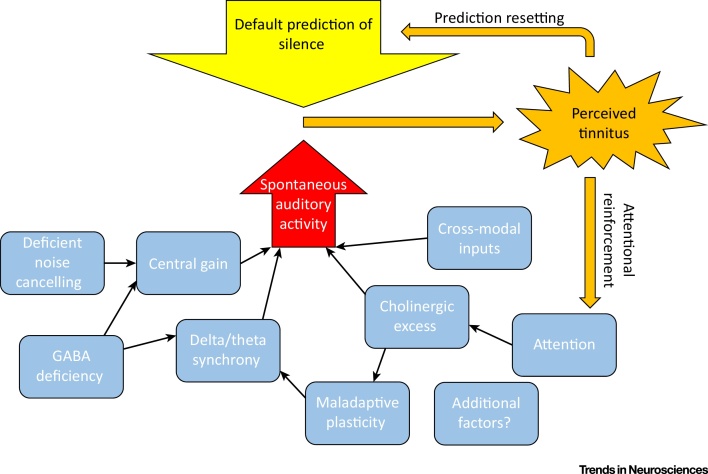
Conceptual Overview of the Model. The core of the model is the process of perceptual inference, driven by a descending auditory prediction (yellow arrow; initially of silence) and by spontaneous activity in the auditory pathway that constitutes a sensory prediction error (red arrow) and, in effect, a ‘tinnitus precursor’. The precision of ascending prediction errors and descending predictions is denoted by arrow width, where the tinnitus precursor has an inherently low precision and therefore makes little or no contribution to perception or posterior beliefs. Various factors (blue boxes) – alone or in combination – can increase the precision of the tinnitus precursor which, if it becomes sufficiently high, results in the perception of tinnitus (orange). Even if this increase in precision is reversed, tinnitus can be perpetuated by two mechanisms (orange arrows): learning to expect tinnitus, and reinforcement via attention.

Under this model, after tinnitus is perceived for a sufficiently long period of time, the default prediction is revised from that of ‘silence’ to that of ‘tinnitus’. Thereafter, even if the precision of the precursor reduces to its pre-tinnitus level, tinnitus is still perceived as the most plausible explanation for the tinnitus precursor. However, the default prediction does not generally encompass the full intensity encoded by the precursor because the precursor is seldom salient or information-rich. A further mechanism of tinnitus self-reinforcement is the direction of attention towards tinnitus, which increases precision via the basal forebrain cholinergic system [59]. A schematic overview of these convergent processes is provided in Figure 1.

## The Neurobiological Model

The tinnitus literature features many proposed underlying mechanisms, almost all of which, we argue, act by increasing the precision of the tinnitus precursor, leading to a predisposition to tinnitus, the onset of tinnitus, or exacerbation of established tinnitus. We summarise these in four categories based on how they affect precision. Crucially, we propose a synergistic (e.g., multiplicative) effect, between changes in each category, on the ultimate impact of the tinnitus precursor on perceptual inference:

(i) The strength of afferent input, for instance neuronal firing rate, conveying the tinnitus precursor to auditory cortex is distinct from the encoding of precision (by synaptic excitability) at the level of auditory cortex, but both encodings have a synergistic effect in terms of their influence on perception. Below the level of auditory cortex, increases in precision at a given level lead to increased firing rates, at higher levels, via postsynaptic gain. Hence, the changes described below can largely be understood either as increased precision of the precursor at specific subcortical levels or as increased intensity at the level of auditory cortex. Increased spontaneous neural firing throughout the central auditory pathway follows noise trauma and tinnitus 19, 60, 61, 62, 63, 64, as does increased central gain 10, 65, 66, 67, which is at least in part due to increased efficacy of somatosensory inputs to the dorsal cochlear nuclei [68]. Increased spontaneous firing in hearing loss can be understood as the consequence of increased postsynaptic gain, which acts to restore mean activity levels. This restoration is mandated predictive coding, where large amplitude prediction errors are attenuated and precise prediction errors are augmented. This implicit normalisation of neuronal activity is the *raison d’être* for PWPE and associated cortical gain control. Neurochemical changes of increased glutamate and reduced GABA have been observed in the auditory brainstem of rats with behavioural evidence of tinnitus [69]; these facilitate transmission of the tinnitus precursor. At a thalamic level, reduced inhibition via the thalamic reticular nucleus (TRN) has been hypothesised, based on structural and functional aberrations in frontostriatal gating circuits that modulate ascending sensory information via the TRN [70]. Hyperpolarised thalamic bursting has been observed in humans [71], and more recently rats [62], with hearing loss and tinnitus.
(ii) The key determinant of precision is the gain of error neurons 36, 38, 50; increasing gain within auditory cortex affords greater precision to subcortical input of a given intensity. Reduced auditory cortex concentration of the inhibitory neurotransmitter GABA has been specifically associated with chronic tinnitus [72]. Focused attention promotes the local release of acetylcholine in auditory cortex via the basal forebrain cholinergic system. Acetylcholine increases the gain on (superficial) pyramidal cells reporting prediction errors [38]. Attention is hypothesised to play a major role in the reinforcement of tinnitus once initiated, and possibly in its initial emergence [59]. Cortical acetylcholine exists in a dynamic equilibrium with, and correlates with the concentration of, total choline [73]. Total choline has been found to correlate with the severity of tinnitus in human patients [72], although a link to acetylcholine remains speculative.
(iii) Synchronous firing of error neurons allows temporal summation of excitatory postsynaptic potentials (EPSPs) on their targets, thereby increasing their influence on postsynaptic responses 51, 52, 74, and in effect their precision. Increased synchrony of nearby single units is observed in animals as a consequence of noise trauma and over-exposure 20, 63, 75, 76, which mirrors the timecourse of behavioural evidence of tinnitus. In humans, gamma oscillations reflect the magnitude and synchrony of superficial pyramidal cell firing 39, 42, and are associated quantitatively with baseline severity of tinnitus [23] and with both short-term 25, 26, 27 and long-term [28] intensity modulations. Hearing loss produces tonotopic map reorganisation [63], leading to abnormally wide cortical areas responding to the same frequency input channel, which may be another factor underlying increased neural synchrony and effective precision, via synchronous gain.
(iv) Low-frequency oscillations are likely to play an important role in modulating the precision of the tinnitus precursor and/or its transmission to higher perceptual areas; however, a specific mechanism is less clear. The magnitude of low-frequency oscillations is increased in tinnitus plus hearing loss 18, 21, and reliably shows suppression alongside short-term suppression of tinnitus using RI 18, 26, 27, 29. Direct human recordings have found that the spatial extent of these oscillations is wide, extending through all of auditory cortex and beyond, and that synchrony between these regions is reduced during tinnitus suppression [27]. While the short time-constants involved mean that low-frequency oscillations are unlikely to have a strong effect on synchronous gain directly, they are known to have a strong modulatory effect on the organisation of high-frequency oscillations 54, 55, and as such may exert indirect effects. One possibility is that they induce long-range synchrony of separate neural populations, generating high-frequency oscillations that project to common targets.

In addition to increasing subcortical gain, cross-modal inputs from orofacial manoeuvres (OFMs) [2] may increase the salience of tinnitus by introducing temporal coincidence between increased tinnitus precursor activity and voluntary movements over a timescale of seconds, thus bestowing behavioural relevance on the signal and increasing precision via top-down influences. We note that this is a much coarser timescale than that underlying long-term potentiation. Alternatively or additionally to contributing to tinnitus emergence, frontostriatal gating mechanisms [70] may also contribute to tinnitus perpetuation or amplification once established, by providing a mechanism via which higher appraisals of stimulus salience can modify the intensity or precision of the precursor at a subcortical level.

The final mechanism, which does not fall into the above categories, relates to maintenance of the tinnitus percept through learning, based on associative plasticity, at higher levels of the auditory and memory systems. This can perpetuate tinnitus once established, even if the precipitating factors acting on precision are removed. While top-down predictions of tinnitus may be generated by various brain regions [33], a strong candidate is the parahippocampal cortex (PHC), which has a role in auditory memory encoding and retrieval. Connectivity between PHC and auditory cortex is increased in tinnitus patients compared to hearing-matched controls 77, 78, and transient tinnitus suppression with RI has been associated with reduced connectivity between auditory cortex and PHC [27]. In addition, resting-state gamma oscillations in PHC are increased contralaterally to perceived tinnitus [79], and a case has been reported of permanent contralateral tinnitus suppression following inadvertent lesioning of connections between PHC and auditory cortex [27].

**Figure 2:**
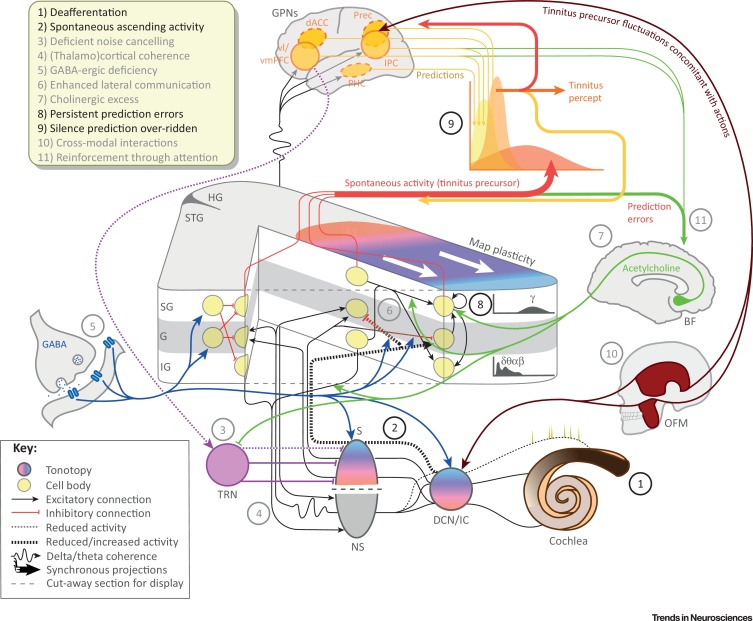
Key Figure: Putative Neurobiological Architecture of the Model

The culmination of the tinnitus precursor, changes in its intensity or precision, and higher predictions (of silence or tinnitus) constitute a process of perceptual inference which, for clarity, we consider at a single crucial pair of levels, comprising auditory cortex as the lower level and higher perceptual networks above. Figure 2 (Key Figure) summarises this process of inference and major contributing mechanisms.

## The Model in Computational Terms

**Figure 3:**
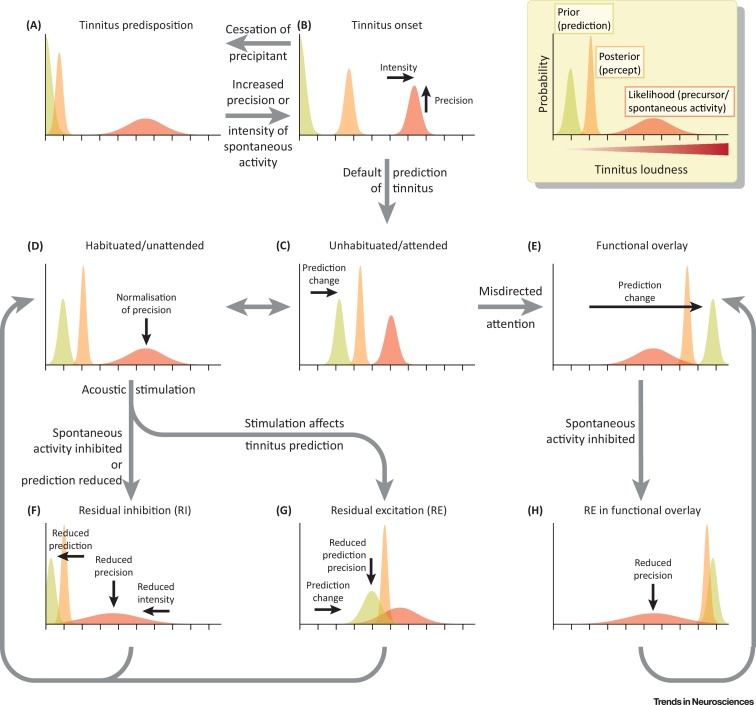
Perceptual Inference Processes Underlying Tinnitus Initiation, Perpetuation, and Modulation. The inset box annotates perceptual inference in terms of the prior (prediction), likelihood (tinnitus precursor) and posterior (tinnitus percept) represented by Gaussian distributions over a perceptual dimension of intensity or loudness, with their widths indicating precision. Loudness may be encoded by neuronal firing rate or be represented more abstractly. In each plot, the perceived loudness of tinnitus is indicated by the position of the posterior distribution on the horizontal axis. (A) In hearing loss alone, the tinnitus precursor has insufficient precision to override the default prediction of silence. (B) With increased precision the precursor influences perception, leading to a revised posterior percept of tinnitus. Potentially there is a window of reversibility at this stage. (C) If the default prediction is revised to expect tinnitus (generally less intense than the precursor), then the condition becomes chronic (through experience-dependent plasticity). (D) Reduction of the precision of the precursor to its pre-tinnitus level results in habituation, but not cessation of tinnitus – on account of plastic changes to prior predictions. (E) Theoretically, patients with functional overlay may have a prediction of louder tinnitus than encoded by the precursor, and therefore tinnitus intensity would have no empirical bound. (F) Residual inhibition (RI) can be understood as attenuating the precision and/or intensity of the precursor through forward masking, thus reducing the precision-weighted prediction error (PWPE) and therefore gamma oscillations. An alternative mechanism is the temporary resetting of descending predictions to ‘silence’, increasing prediction error *per se* (hence gamma) but reducing the posterior percept. (G) In residual excitation (RE), temporary modification of the tinnitus prediction (increasing its loudness and/or reducing its precision) by a perceptually similar and precise stimulus leads to reduced prediction error (hence gamma), and increased tinnitus loudness more in line with that encoded by the precursor. (H) In functional overlay patients, acoustic forward masking, and the consequent fall in gamma oscillations, bias inference towards higher tinnitus intensity than encoded by the precursor (leading to RE).

This section considers the computations within the perceptual inference framework that give rise to the onset, perpetuation, habituation, and RI and RE of tinnitus. It also explains the paradoxical neural correlates associated with RI and RE [26], and the theoretical circumstances in which the intensity of the tinnitus prediction can exceed that of the tinnitus precursor, together with their perceptual and neurophysiological consequences. While qualitative, this specification of the model is theoretically amenable to quantitative computational modelling of simulated and/or empirical data. Figure 3 illustrates and explains the situations listed above. A key feature of this sequence of events is that changes to factors affecting the precision or intensity of the tinnitus precursor, which are responsible for the neural correlates associated with hearing loss and tinnitus, need only be transiently elevated compared to the baseline state of predisposition to tinnitus. Transient elevation in precision may occur as a result of neurophysiological, hormonal, and/or neurochemical factors, including attention, illness, and stress. Once tinnitus is established, these factors can return to baseline levels, offering an explanation for why tinnitus patients and matched controls have not been conclusively found to differ in any of these correlates. In fact, resetting of the default prediction should actually lower ongoing prediction errors, if anything leading to slightly reduced gamma oscillations compared to matched controls. Importantly, the model is also compatible with persistent changes in one or more of these factors – but no such persistent change is required.

The amplitude of gamma oscillations in the auditory cortex reflects PWPE 40, 41, and is therefore positively influenced by **prior precision**, likelihood precision, and prediction error (difference between prior and likelihood means). Thus, gamma oscillations can be treated as a proxy for the amplitude of prediction errors where precisions are fixed – or the sensory or prior precision where prediction errors are fixed. Because the prior generally predicts a less intense or loud tinnitus than the precursor, an inverse correlation between tinnitus loudness and gamma magnitude can arise in the following circumstances: reducing prior precision reduces the PWPE (hence gamma) while skewing perception towards the (louder) tinnitus precursor; increasing the loudness/intensity of the prior also increases the loudness of the posterior percept, but reduces prediction error and therefore gamma magnitude.

A fundamental tenet of predictive coding is the minimisation of total PWPE, across all hierarchical levels [80]. In some circumstances, maintaining an unresolved prediction error at one hierarchical level can resolve discrepancies (prediction errors) at other levels. In our model, such a persistent prediction error is maintained by the prior intensity of tinnitus remaining lower than the intensity of the precursor. This has several implications:

(i) Auditory cortex gamma oscillations, a signature of PWPE, remain elevated, rather than being resolved by top-down predictions (which would otherwise lead to reduced gamma oscillations compared to control subjects).
(ii) Attention increases the gain on auditory prediction errors, and therefore increases the perceived intensity of tinnitus [59].
(iii) The paradoxical gamma oscillation findings in RE [26] can only occur in the context of this type of hierarchical PWPE minimisation, leaving an unresolved prediction error in at least one interacting pair of levels.
(iv) There is generally an upper bound on tinnitus intensity, although generally tinnitus is perceived as less intense than this limit. Theoretically, the intensity of tinnitus could exceed that of the precursor. This could arise for reasons such as catastrophising, or misdirected attention (as is theorised to occur in **functional** neurological patients [81]) leading to a ‘functional overlay’. Thus, a subgroup of patients is predicted to exist, who may experience other functional physical symptoms, have some associated personality traits, report extraordinarily intense tinnitus, and have high levels of tinnitus distress. In addition, in such patients, acoustic forward masking of the tinnitus precursor (which usually gives rise to RI) could be associated with exacerbations in tinnitus loudness (RE), and the neural correlates usually associated with RI.

## Closing Remarks

The new model joins a family of predictive coding-based accounts of positive perceptual disorders, including schizophrenia [82], in which reduced prior precision – or a failure to attenuate sensory precision – is posited as a basis for false perceptual inference, and musical hallucinosis [8] in which high-intensity low-precision sensory activity is shaped into music by relatively precise priors. The present model differs from these in that it postulates excessive sensory precision as the basis for perception of a ‘real’ but usually imprecise sensory signal. This crucial difference explains the relatively simple and unchanging content of tinnitus, which must remain largely yoked to the characteristics of the tinnitus precursor, as opposed to the florid and structured perceptual experiences in musical hallucinosis and schizophrenia, which are dynamically shaped by a range of brain centres involved in complex perception and imagery.

The new model offers a framework into which existing tinnitus theories and models contribute synergistically, without mutual exclusivity or requiring alternative mechanisms for a single clinical syndrome [32]. The validity of the new framework does not depend upon any specific contributing mechanism, and future refinements may include removal of some of these and/or inclusion of others. Importantly, the model resolves all the tinnitus paradoxes described in Box 1 – where existing theories alone or in combination cannot (Box 1, Table I).

The model we have introduced, comprising peripheral and/or central subcortical sources of spontaneous sensory input and their hierarchical processing in a predictive coding framework – in which perception is heavily shaped by precision and higher predictions – is unlikely to be unique to tinnitus. While there are subcortical structures specific to the auditory system, the broader framework is equally applicable to other conditions characterised by chronic low-level sensations. These might include conditions often compared to tinnitus, such as central or neuropathic pain, and more common scenarios such as chronic nociceptive pain. Resetting of default predictions could help to explain persistent pain following healing of the initiating trauma, if normal ongoing sensory activity (a ‘pain precursor’) is enough to ‘activate’ established predictions. Misdirected attention (functional overlay) could lead to amplification of spontaneous sensory activity, beyond the intensity of the ‘pain precursor’, to cause chronic pain in the absence of peripheral nociceptive stimulation. However, there are types of central pain, such as central post-stroke pain [83], that are not easily explicable by our model.

Finally, the model generates avenues for future research and presents several testable hypotheses (see the Outstanding Questions).

Outstanding Questions We propose that transient changes in precision of the precursor are responsible for the onset of tinnitus. This leads to the testable hypothesis that neural signatures relating to increases in precision (postsynaptic gain) are elevated at around the time of tinnitus onset, and return to approximately pre-tinnitus levels with habituation.

All neural processes in the model, except the origin of the precursor, are not specific to subjective tinnitus, but are responses to chronic subcortical noise-like stimulation, which could be studied using a chronic external stimulus or ‘pseudo-tinnitus’. If the phenomenology and neural correlates of tinnitus can be reproduced in the context of pseudo-tinnitus, this would bring several benefits, including excellent matching of ‘control’ subjects, equivalence of animal and human methods, reversibility, and the ability to study onset and development in humans.

The model argues that currently suggested neural correlates of ‘tinnitus’ are those of predisposing factors or disequilibrium in the system. Future work might be able to indirectly expose the representational content (i.e., tinnitus and its characteristics) of the system.

If oscillatory correlates relating to tinnitus and hearing-loss reflect the predisposition to tinnitus rather than the condition itself, then acoustic forward masking (with stimuli that produce RI in tinnitus subjects) in hearing-matched controls should result in the same oscillatory activity changes.

The model predicts potential avenues for treatment, including early intervention during a window of reversibility following perceptual tinnitus onset, manipulation of the precision of the tinnitus precursor, and disrupting the circuitry (e.g., auditory–parahippocampal connections) that maintains the tinnitus prediction.

## Figures and Tables

**Figure I:**
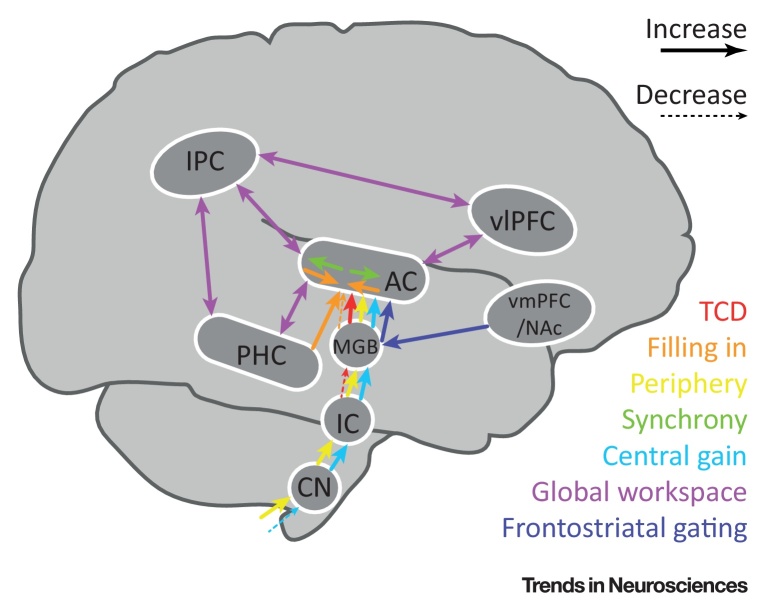
Schematic of Altered Inter-Areal Inputs in Existing Models of Tinnitus Generation.

**Table I:** Comparison of Current and Existing Tinnitus Models in Terms of Ability to Address Paradoxes in Tinnitus Research^a^

	Improvement by auditory nerve section	Exacerbation by auditory nerve section	Occurrence of hearing loss without tinnitus	Onset of tinnitus later than hearing loss	Lack of spontaneous neural correlates in patients versus matched controls	Bidirectional correlation of tinnitus intensity with gamma power
Peripheral	Yes	No	Potentially	Potentially	No	No
Central gain	No	Yes	Potentially	Potentially	No	No
Neural synchrony	No	Yes	Yes	Yes	No	No
Thalamocortical dysrhythmia	No	Yes	Potentially	Potentially	No	No
Frontostriatal gating	No	Yes	Yes	Potentially	No	No
Filling in	No	Yes	No	Potentially	Potentially	No
Global workspace	N/A	N/A	Yes	Yes	Potentially	No
Precision/predictive coding model	Yes: (i) If precursor origin is peripheral (ii) Deafferentation reduces sensory precision	Yes: (i) If precursor origin is central (increased central gain) (ii) Deafferentation removes constraints on sensory precision	Yes: Adaptive attenuation of sensory precision	Yes: Failure to attenuate sensory precision, which is influenced by many factors, including stress	Yes: Neural correlates of PWPE reflect predisposition to tinnitus rather than to the percept itself	Yes: Hierarchical dissociation of perceptual inference (tinnitus) and concomitant PWPE (gamma)

a Yes, addresses paradox; No, cannot address paradox; Potentially, does not presently address paradox but could do so with amendment; N/A, not applicable.

## Box 1 Summary of Existing Tinnitus Models and Their Limitations

Peripheral models [12] explain tinnitus as the consequence of aberrant cochlear activity. They can explain the observed correlation between central and peripheral neuronal firing [12], and tinnitus alleviation by cochlear suppression or auditory nerve section [16], but not exacerbation by auditory nerve section [16] or ear plugging [14], nor the late emergence of tinnitus during intercurrent stress.

In subcortical hyperactivity models, tinnitus is caused by excessive spontaneous subcortical neural activity relayed to auditory cortex. These include central gain 10, 84, somatosensory–auditory interactions 85, 86, frontostriatal gating 70, 87, and thalamocortical dysrhythmia 31, 34. They can explain the emergence of tinnitus through hearing loss, ear plugging, and exacerbation by auditory nerve section, but not alleviation by auditory nerve section, or the lack of a neural correlate distinguishing patients from hearing-matched controls [18].

In neural synchrony models 20, 75, excessive local synchrony of neuronal firing, originating at or below the level of auditory cortex, is responsible for tinnitus. Tonotopic map plasticity [63], by which over-representation of particular frequencies could lead to excessive synchrony, fall within this category. As with hyperactivity models, there is a lack of neural correlates distinguishing patients from controls.

Focused attention, acting via the basal forebrain cholinergic system, does not explain the origin of the tinnitus signal, but may play an important role in explaining phenomena such as habituation, and emergence during intercurrent stress [59].

In filling-in models 31, 59, 88, deafferented parts of auditory cortex receive reduced or absent subcortical input, and therefore obtain their input from adjacent normally-functioning cortex or auditory memory retrieval. These suffer from a face validity problem because they predict that the tinnitus percept should resemble current or recent auditory input or familiar auditory percepts, whereas tinnitus is generally an unchanging, low-level, unfamiliar, percept. Furthermore, these models assume that spontaneous subcortical input is reduced in hearing loss, whereas it is actually increased.

Global workspace models [33] do not specify the origin of tinnitus, but invoke wider brain networks to explain its conscious perception as well as consequences such as distress and autonomic reactions.

Collectively, these models (Figure I) are problematic because (i) they require a ‘multiple origins’ framework, (ii) elevated spontaneous auditory cortex activity or synchrony in tinnitus patients compared to hearing-matched controls has not been demonstrated, and (iii) except for global workspace, each model contradicts at least one other, in terms of one or more aberrant changes in local activity or connectivity.

Table I summarises the shortcomings of existing models, and how these are solved by the new model.

## Glossary

Cause: object or event causing sensory input. In predictive coding, internal models hold priors about causes, and these are combined with sensory evidence to generate posteriors that provide predictions of sensory input.
Central: refers to neural pathways within the brain, from the level of cranial nerve nuclei upwards.
Functional: physical, often neurological, symptoms in the absence of, or in excess of, physical dysfunction of the related organ system. Alternative (non-preferred) terms include ‘hysterical’, ‘psychosomatic’, and ‘conversion’.
Habituation: process by which, over time, people experience reduced awareness, intrusiveness, and/or loudness of tinnitus. In this article we focus particularly on reduced loudness.
Likelihood: probability of a sensory input given a specific cause (e.g., tinnitus or silence). Ascending sensory information or prediction error reports the likelihood of a cause.
Oscillations: periodic fluctuations in electromagnetic field/potential as a result of synchronised firing of neuronal ensembles. Categorised by approximate frequency band (repetition rate): delta, 1–4 Hz; theta, 3–8 Hz; alpha, 8–12 Hz; beta, 12–30 Hz; gamma, >30 Hz.
Peripheral: (auditory) pathways outside the brain including external and middle ear, cochlea, and auditory nerve.
Posterior: the product of perceptual inference or predictive coding. Probability distribution over the causes of data (sensory input), after their observation. The posterior expectation is a mixture of the likelihood and prior expectations, each weighted by its precision.
Precision: reliability of or confidence in a belief or neural representation (i.e., prior or likelihood), which determines its influence on the posterior. Mathematically, precision is the inverse of variance.
Prediction: predicted consequences (e.g., sensory input) based upon posterior expectations of underlying causes. Predictions are thought to be conveyed by descending connections.
Prediction error: mismatch between sensory input and prediction (sensory prediction error), or between expectation at one hierarchical level and prediction from the level above (prior prediction error).
Predictive coding: an account of brain function explaining perception as the posterior product of prior predictions and sensory input (or likelihood), each weighted by its precision. Bottom-up connections convey prediction errors that are communicated via gamma oscillations, while top-down connections convey predictions and involve beta oscillations.
Prior: part of a generative model specifying a belief over the causes of consequences (e.g., sensory input) before their observation.
Prior precision: precision or confidence afforded a prior belief.
Residual excitation (RE): process by which tinnitus temporarily becomes louder after an acoustic stimulus; typically a narrowband stimulus of similar spectral frequency to the tinnitus.
Residual inhibition (RI): process by which tinnitus is masked by an external acoustic stimulus, and remains temporarily suppressed after that stimulus has ended.
Sensory precision: precision or confidence of a likelihood or sensory prediction error. Neuronally, this corresponds to the gain of principal cells that are the source of ascending sensory prediction errors.
